# Efficient Chemical Sensing by Coupled Slot SOI Waveguides

**DOI:** 10.3390/s90201012

**Published:** 2009-02-16

**Authors:** Vittorio M. N. Passaro, Francesco Dell'Olio, Caterina Ciminelli, Mario N. Armenise

**Affiliations:** Dipartimento di Elettrotecnica ed Elettronica, Politecnico di Bari, via Edoardo Orabona n. 4, 70125 Bari, Italy

**Keywords:** Integrated optics, Optical sensor, Slot waveguides, Silicon-on-insulator

## Abstract

A guided-wave chemical sensor for the detection of environmental pollutants or biochemical substances has been designed. The sensor is based on an asymmetric directional coupler employing slot optical waveguides. The use of a nanometer guiding structure where optical mode is confined in a low-index region permits a very compact sensor (device area about 1200 μm^2^) to be realized, having the minimum detectable refractive index change as low as 10^-5^. Silicon-on-Insulator technology has been assumed in sensor design and a very accurate modelling procedure based on Finite Element Method and Coupled Mode Theory has been pointed out. Sensor design and optimization have allowed a very good trade-off between device length and sensitivity. Expected device sensitivity to glucose concentration change in an aqueous solution is of the order of 0.1 g/L.

## 1. Introduction

In recent years considerable research effort has been developed to employ electronic and optical micro- and nano-sensors in a great number of application fields such as medicine, microbiology, particle physics, automotive, environmental safety and defence. Sensor and actuator monolithic integration in micro-electro-mechanical systems (MEMSs) and micro-opto-electro-mechanical systems (MOEMSs) has been achieved in a large number of sensing devices. Simultaneously, photonic sensors have attracted great attention because of their immunity to electromagnetic interference, good compactness and robustness and high compatibility with fibre networks, but also due to the shorter response time and higher sensitivity and stability possible, compared to MEMS/MOEMS devices.

A number of peculiar characteristics are usually required for electronic and photonic sensors used for chemical applications, such as biomolecule concentration measurement, DNA sequencing, pH estimation, pollutant control and gas monitoring. A chemical sensor has to be contextually highly sensitive and selective to the analyte being detected, as well as immune to external disturbances such as pressure or temperature changes. Using integrated photonic technologies, it is possible to fabricate very compact, high performing and low-cost chemical and biochemical sensors [1]. Different kinds of integrated optical chemical sensors have been proposed over the years, like those based on directional couplers [2], Mach-Zehnder interferometers [3-5], Bragg gratings [6-7], micro-ring resonators [8-10], and photonic crystal micro-cavities [11]. Many of these devices have been realized adopting CMOS-compatible technological processes.

Among the CMOS-compatible technologies, Silicon-on-Insulator (SOI) is emerging as the most attractive for a wide spectrum of applications [12]. The reasons of this great interest in SOI-based integrated optics are related to the possibility to realize waveguides exhibiting low loss (less than 1 dB/cm) and high index contrast, the low cost of silicon and wide production infrastructure available for silicon based integrated device fabrication. SOI technological platform has been demonstrated as very attractive in the realization of highly compact integrated optical structures, such as micro-ring and micro-racetrack resonators [13], Bragg gratings [14], and Fabry-Perot microcavities [15].

**Figure 1.**
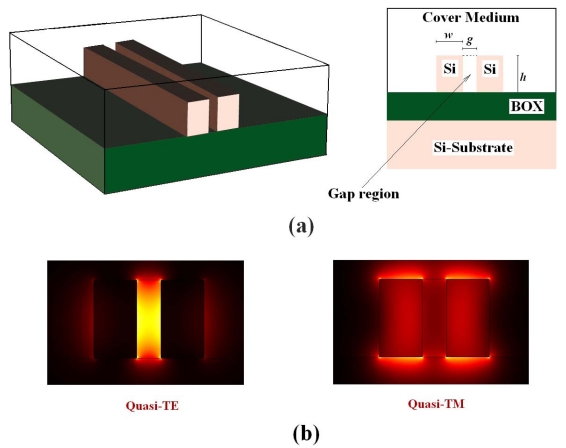
(a) Slot waveguide typical structure (BOX: Buried Silicon Oxide). (b) Profiles of quasi-TE and quasi-TM modes confined in a slot waveguide.

There is a current trend in SOI photonic integrated circuits and devices to move toward smaller dimensions to achieve cost efficiency and for device performance improvement. In this scenario, a silicon nanometer guiding structure, usually referred to as a slot waveguide [16], is attracting considerable attention. In optical slot waveguides, e.g. fabricated in SOI technology by either e-beam or deep-UV lithography [17], the electric field discontinuity at the interface between high index contrast materials enables high optical confinement inside a nanometer-scale area (gap region) of low-index material (e.g. air, silicon oxide, aqueous solutions, silicon nanocrystals, thermo-optic or electro-optic polymers). This guiding structure can be realized nearing two silicon wires having nanometer dimensions, as in Figure 1(a). It supports both fundamental quasi-TE and quasi-TM modes, whose typical profiles are shown in Figure 1(b). The quasi-TE mode is highly confined in the low-index gap region between the wires, whereas only about 5 % of the optical power carried out by the quasi-TM mode is localized in the gap region.

In the last three years, a great variety of optical devices have been proposed and realized using SOI slot waveguides, including micro-ring resonators [18-20], disk resonators [21], optical micro-switches [22] and modulators [23-25], nano-mechanical photonic sensors [26], electrically pumped light emitting devices [27], directional couplers [28-29], all-optical logic gates [30], multimode interference beam splitters, wavelength demultiplexers [31-32], polarization rotators [33] and one dimensional photonic crystals [34-35].

Very recently, optical manipulation of nanoparticles and DNA molecules has been successfully demonstrated in silicon slot waveguides [36]. Consequently, the use of slot waveguides in lab-on-a-chip micro-systems is becoming more and more attractive.

The non linear properties of slot waveguides have been also investigated assuming the gap region filled by highly non linear materials, e.g. silicon nanocrystals [37-39]. Gaussian-like mode coupling into a slot waveguide has been achieved with a loss around 0.01 dB with a 100 μm long integrated device [40]. Grating couplers for SOI slot waveguides have been theoretically investigated too [41].

Alternative materials such as silicon nitride or Al_0.3_Ga_0.7_As have been recently proposed for slot guiding structures [42]. Horizontal slot waveguides have been also optimized [43], experimentally investigated [44] and employed to enhance photoluminescence of Er^3+^ doped Si nanoclusters in a low-index SiO_2_ matrix [45]. A multiple-slot guiding structure has been proposed to further enhance the optical confinement inside the slot regions [46]. To decrease the group velocity of an optical wave propagating in a slot waveguide, a silicon-based slot photonic crystal guiding structure has been proposed and demonstrated [47]. An electro-optic modulator based on a similar structure has been theoretically analyzed [48]. Finally, plasmonic slot waveguides based on a deep nanometer slot in a thin metallic film have been widely investigated for their use at visible and near infrared wavelengths [49].

The slot guiding structure effective index is very sensitive to changes in the cover medium refractive index. Adopting as cover medium an aqueous solution, whose refractive index depends on the concentration of the analyte of interest (glucose or ethanol, for example), we could observe a waveguide effective index shift due to analyte concentration changes. By monitoring the slot guiding structure effective index in an appropriate integrated architecture (e.g. Mach-Zehnder interferometer, ring resonator, Bragg grating, directional coupler, and so on), it is possible to fabricate highly sensitive and miniaturized integrated optical chemical sensors.

Slot waveguides have recently been demonstrated as very useful for chemical and biochemical optical sensing [50] and an integrated biochemical sensor (operating at 1,300 nm) based on a slot-waveguide microring resonator in Si_3_N_4_/SiO_2_ has been also demonstrated [51]. Highly sensitive molecular binding detection by a Si_3_N_4_/SiO_2_ slot waveguide has been experimentally demonstrated in [52]. Finally, a micro-ring resonator exploiting a SOI slot guiding structure has been adopted for gas sensing [53].

In this paper, we propose the use of slot waveguides to design an asymmetric directional coupler for optical chemical sensing. SOI technology has been assumed in the design at operating wavelength λ = 1,550 nm. Finite Element Method (FEM) [54] and Coupled Mode Theory (CMT) have been employed for device modelling and optimization. CMT has been previously adopted for designing symmetrical directional couplers based on SOI slot waveguides [29]. Model validation by an accurate simulation tool based on 3D EigenMode Expansion (EME) method [55] has been also performed.

## 2. Sensor architecture and guiding structure

**Figure 2.**
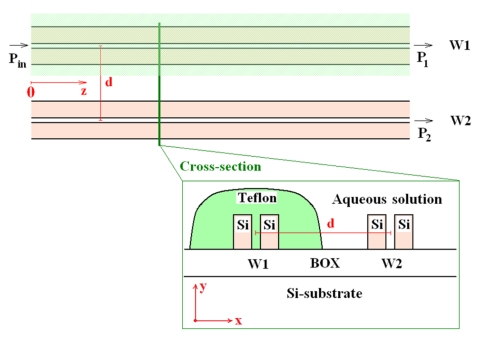
Architecture of proposed integrated optical sensor.

The proposed integrated sensor, whose architecture is shown in Figure 2, includes two parallel slot waveguides separated by a distance *d* between slot waveguide centres, then forming a directional coupler.

This coupler is asymmetric because in one guiding structure (*W1*) the cover material (also filling the gap) is Teflon fluoropolymer (refractive index 1.31) whereas in the other waveguide (sensing waveguide, *W2*) the cover medium is constituted by an aqueous solution whose refractive index (around 1.33) depends on the concentration of the chemical agent (e.g. glucose) dispersed in the solution. In fact, a glucose concentration change of 1 g/L induces a refractive index shift in the solution of the order of 10^-4^ [56].

In principle, different materials other than Teflon could be used as cover medium in *W1*, i.e. silicon dioxide (refractive index 1.444). However, in this case Teflon is an appropriate choice because its refractive index is quite near to the refractive index of the aqueous solution (the difference is around 1.5 %). In this way, the asymmetry in the directional coupler is moderate. Technological aspects of Teflon deposition on silicon have been widely discussed in [57].

When the concentration changes, a shift in refractive index of *W2* cover occurs. This variation has been assumed ranging from 1.333 to 1.334, i.e. of the order of 10^-3^, corresponding to a glucose concentration change of about 10 g/L [56]. In turn, this shift induces a variation of optical power coming out from both waveguides, because a change of coupling conditions between the two optical modes propagating in the coupled slot waveguides occurs. Measuring the optical powers at the output of the two waveguides and/or their difference by two photo-detectors, it is possible to estimate with high sensitivity the concentration of the chemical agent dispersed in the solution. In fact, in a generic slot waveguide, the sensitivity *S_w_* to any cover medium refractive index change can be defined as:

(1) $$S_{w}=\frac{\partial n_{\text{eff}}}{\partial n_{c}}$$

where *n_eff_* is the effective index of the excited optical mode propagating in the slot waveguide and *n_c_* is the covering substance refractive index. This parameter depends on the electric field squared module fraction confined in the cover and, according with variational theorem for dielectric waveguides, it can be given by:

(2) $$S_{w}≅\frac{2n_{c}^{0}}{\eta_{0}P}{\iint_{C}\left|E\left(x,y\right)\right|}^{2}=\frac{2n_{c}^{0}}{\eta_{0}}\Gamma_{C}\cdot \Pi$$

where

(3) $$\Gamma_{C}=\frac{{\iint_{C}\left|E\left(x,y\right)\right|}^{2}}{{\iint_{\infty }\left|E\left(x,y\right)\right|}^{2}}$$

and

(4) $$\Pi =\frac{{\iint_{\infty }\left|E\left(x,y\right)\right|}^{2}}{P}$$

*n_c_^0^* is the unperturbed value of the cover refractive index, *η_0_* is the free space impedance, *P* is the optical power carried by the propagating mode, *C* indicates the cover medium region (including also the gap region), and **E** is the electric field vector relevant to propagating mode (being *x* and *y* the transverse coordinates). Quasi-TE mode supported by a slot waveguide is highly confined in the gap region, as in Figure 1(b), and its confinement factor (*Γ_C_*) in the cover is typically around 60-70%, which means a very large sensitivity to cover index change. Quasi-TM mode is significantly less sensitive to cover index change and its confinement factor *Γ_C_* is only around 40-50%. SOI slot waveguide sensitivity has been already optimized by a very accurate FEM-based approach [50]. A guiding structure having a sensitivity *S_w_* = 1.0076 for quasi-TE mode has been demonstrated, assuming silicon and silicon oxide refractive index as equal to 3.476 and 1.444, respectively. The theoretical possibility that sensitivity of a guiding structure could be larger than one has been firstly predicted in [58]. Dimensions of the slot waveguide optimized in [50] have been also used in this paper (summarized as in Table 1). Sensitivity (for quasi-TE mode) of SOI slot waveguide in our design is more than two times larger than that calculated for Si_3_N_4_/SiO_2_ slot waveguide adopted in the sensor reported in [51] (having *S_w_* = 0.453), using the modelling technique developed in [50].

## 3. Sensor modelling

The directional coupler-based integrated sensor has been modelled by CMT developed for generic monomodal parallel waveguides [59]. Electric field amplitudes (*A_1_* and *A_2_*) of two co-propagating modes can be calculated by the following differential equations:

(5) $$\left\{ \begin{array}{l} \frac{dA_{1}}{dz}=i\kappa_{11}A_{1}+i\kappa_{12}A_{2}e^{i\frac{2\pi }{\lambda }\left(n_{\text{eff},2}-n_{\text{eff},1}\right)z}\\ \frac{dA_{2}}{dz}=i\kappa_{21}A_{1}e^{i\frac{2\pi }{\lambda }\left(n_{\text{eff},1}-n_{\text{eff},2}\right)z}+i\kappa_{22}A_{2} \end{array}\right.$$

where *z* is the propagation direction, *κ_ij_* (*i*, *j* = 1, 2) are the coupling coefficients, and *n_eff,1_* and *n_eff,2_* are the effective indices of modes propagating in the two isolated slot waveguides. Assuming the input optical power as launched in *W1* (see Figure 2), the normalized optical power coming out from *W1* and *W2* can be written as [59]:

(6) $$P_{1}=\Psi^{2}\frac{\kappa^{2}}{\beta_{c}^{2}}{\left[\cos\left(\beta_{c}L\right)\right]}^{2}+\Psi^{2}\frac{\delta^{2}}{\beta_{c}^{2}}$$

(7) $$P_{2}=\Psi^{2}\frac{\kappa^{2}}{\beta_{c}^{2}}{\left[\sin\left(\beta_{c}L\right)\right]}^{2}$$

where

(8) $$\beta_{c}=\sqrt{\kappa^{2}+\delta^{2}}$$

(9) $$\kappa^{2}=\kappa_{12}\kappa_{21}$$

(10) $$\delta =\frac{\pi }{\lambda }\left(n_{\text{eff},2}-n_{\text{eff},1}\right)+\frac{\kappa_{22}-\kappa_{11}}{2}$$

*Ψ* is the coefficient taking into account the electric field amplitude attenuation due to optical losses and *L* is the directional coupler length. Difference between self-coupling coefficients *κ*_11_ and *κ*_22_ is equal to:

(11) $$\begin{array}{l} \kappa_{22}-\kappa_{11}=\left[\frac{2\pi c}{\lambda }\left(n^{2}\left(x,y\right)-n_{1}^{2}\left(x,y\right)\right)\iint_{W2}{\left|E_{1}\left(x,y\right)\right|}^{2}\text{dxdy}\right]-\\ -\left[\frac{2\pi c}{\lambda }\left(n^{2}\left(x,y\right)-n_{2}^{2}\left(x,y\right)\right)\iint_{W1}{\left|E_{2}\left(x,y\right)\right|}^{2}\text{dxdy}\right] \end{array}$$

where *c* is the free space light speed, *n*(*x*,*y*) is the refractive index distribution in the coupler cross section, *n_1_*(*x*,*y*) and *n_2_*(*x*,*y*) are the refractive index distributions relevant to isolated slot guiding structures, and **E_1_**, **E_2_** are the normalized electric fields of optical modes propagating in isolated *W1* and *W2*, respectively. If both co-propagating modes are well confined in their guiding structures, the difference between self-coupling coefficients is negligible when distance between the waveguides is sufficiently large, because the two integrals in Eq. (11) tend to vanish (being **E_1_** squared module integrated in *W2* region and **E_2_** squared module integrated in *W1* region).

**Figure 3.**
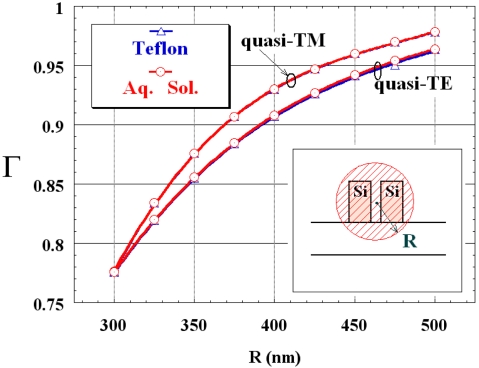
Confinement factor in a circular region versus *R* (cover medium: Teflon or aqueous solution).

To calculate the distance *d* between the waveguides in order to achieve a negligible difference between self-coupling coefficients in Eq. (10), the confinement factor *Γ* in a circle having a radius *R* and centred at the gap region centre has been evaluated for the single slot waveguide (see inset in Figure 3). Dependence of *Γ* on *R* is sketched in Figure 3 for quasi-TE and quasi-TM modes, when the cover medium is either Teflon or an aqueous solution. For *R* values in the range 300 nm < *R* < 500 nm, *Γ* is practically independent from cover refractive index, being larger than 90 % for *R* ≥ 400 nm, for both polarizations.

**Figure 4.**
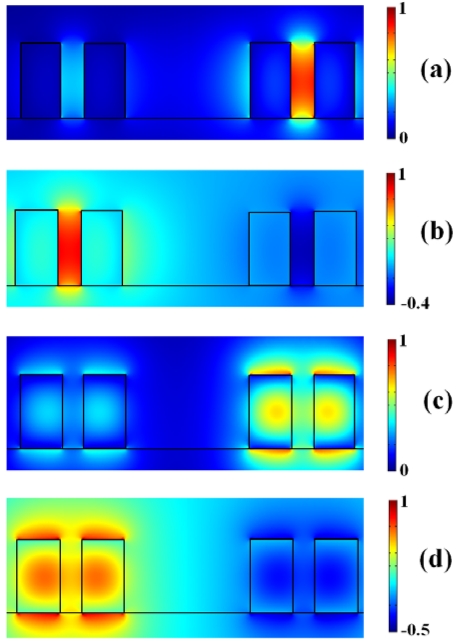
Main electric field component distribution related to supermodes supported by the asymmetrical directional coupler (*d* = 1 μm). (a) Quasi-TE symmetric supermode. (b) Quasi-TE antisymmetric supermode. (c) Quasi-TM symmetric supermode. (d) Quasi-TM antisymmetric supermode.

For a distance *d* ≥ 800 nm between the coupled waveguides, both integrals in Eq. (11) are practically negligible because **E_1_** squared module in *W2* region and **E_2_** squared module in *W1* region are very close to zero. Then, for *d* ≥ 800 nm the second additive term in Eq. (10) is negligible, and *δ* can be estimated by calculating *n_eff,1_* and *n_eff,2_* through the 2D FEM applied to the two isolated slot waveguides. According with CMT, *β_c_* in Eq. (8) can be calculated as [59]:

(12) $$\beta_{c}=\frac{\pi }{\lambda }\left(n_{\text{eff},S}-n_{\text{eff},A}\right)(12)$$

where *n_eff,S_* and *n_eff,A_* are the effective indices of the symmetric (*S*) and antisymmetric (*A*) supermodes supported by the directional coupler (supermodes profiles are in Figure 4). Supermodes effective indices *n_eff,S_* and *n_eff,A_* have been again calculated by 2D full-vectorial FEM applied to the coupler cross-section. Finally, *κ*^2^ has been calculated as:

(13) $$\kappa^{2}=\beta_{c}^{2}-\delta^{2}$$

**Figure 5.**
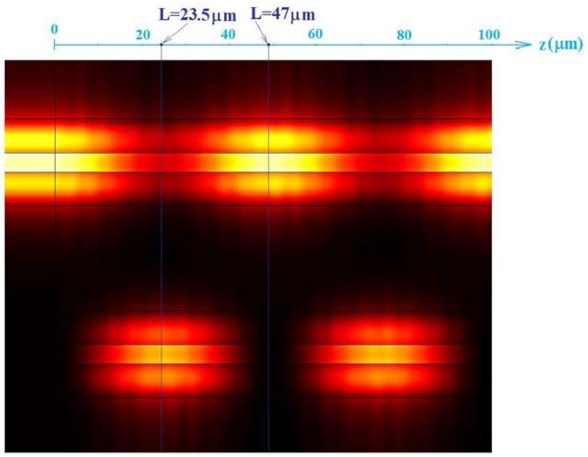
Quasi-TE mode optical propagation in the designed asymmetrical directional coupler (*d* = 1 μm). Optical field intensity plot obtained by EME method.

FEM is widely adopted in calculation of mode effective indices because it assures a very high accuracy even for nanometer-scale high index contrast guiding structures. To validate the proposed modelling technique, the asymmetrical directional coupler behaviour has been 3D simulated by EME method [55] in the more sensitive case of quasi-TE polarization. Geometrical parameters reported in Table 1 have been assumed for slot waveguides constituting the coupler with *d* = 1 μm. In this simulation, cover refractive index is equal to 1.333 for sensing waveguide (*W2* in Figure 2), and 1.31 for the other one (*W1* in Figure 2). Simulation of optical propagation within the coupler performed by 3D EME method is shown in Figure 5.

**Figure 6.**
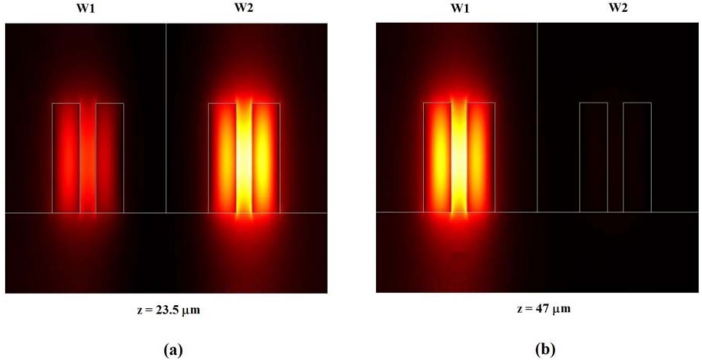
Optical intensity (quasi-TE mode by EME method) distribution in the cross-section of asymmetrical directional coupler (*d* = 1 μm) at: (a) *z* = 23.5 μm; (b) *z* = 47 μm.

**Figure 7.**
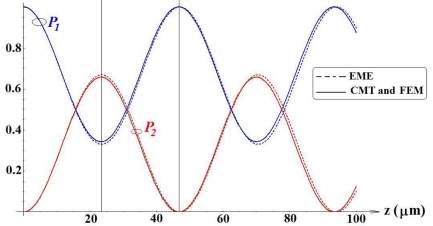
Normalized optical power confined in the two coupled waveguides (with *d* = 1 μm) versus propagation length for quasi-TE mode (Solid curves: modelling technique of this paper based on CMT and FEM; Dashed curves: results by 3D EME method).

At the section *z* = 23.5 μm we observe the maximum power transfer from *W1* to *W2* [see Figure 6(a)], whereas at *z* = 47 μm the whole optical power is practically confined in *W1*. The optical intensity distribution in the coupler cross-section for *z* = 47 μm is shown in Figure 6(b). For *z* = 23.5 μm, normalized optical powers confined in *W1* and *W2* have been estimated as 0.3302 and 0.6698, respectively. For *z* = 47 μm, normalized powers confined in *W1* and *W2* are equal to 0.9724 and 0.0276, respectively. Normalized optical powers confined in *W1* (*P_1_*) and *W2* (*P_2_*) have been also calculated versus propagation direction *z* by Eqs. (6)–(7) (see Figure 7). The agreement between simulation results obtained by EME method and our modelling procedure is very good, as in Figure 7. From our calculations, coupling length between *W1* and *W2* is accurately predicted to be *z* = 23.33 μm.

## 4. Sensor optimization and performance

**Figure 8.**
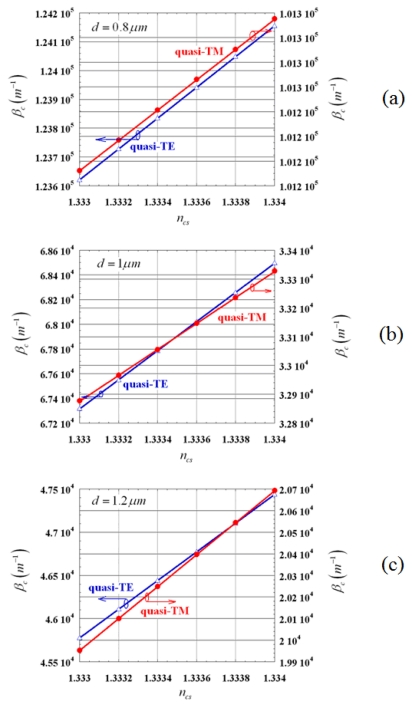
Dependence of *β_c_* on *n_cs_*: a) *d* = 0.8 μm; b) *d* = 1 μm; c) *d* = 1.2 μm.

For a given directional coupler length *L*, the normalized optical power coming out from both waveguides depends on the sensing waveguide (*W2*) cover index *n_cs_* (1.333 ≤ *n_cs_* ≤ 1.334) and distance *d* between the guiding structures. For *d* = 0.8 μm, 1 μm, 1.2 μm, *β_c_* dependence on *n_cs_* is sketched in Figure 8 for quasi-TE and quasi-TM modes.

In all considered cases, *β_c_* increases with increasing *n_cs_*. The ratio between *β_c_* values related to quasi-TE and quasi-TM modes is around 1.2 for *d* = 0.8 μm, 2 for *d* = 1 μm and, 2.2 for *d* = 1.2 μm. This implies that *P_1_* varies more rapidly for quasi-TE than for quasi-TM as *L* changes (see Eq. (6)). *β_c_* dependence on *n_cs_* is highly linear, so we can get the following formula:

(14) $$\beta_{c}=q_{0}+q_{1}n_{cs}$$

where *q_0_* and *q_1_* are fitting parameters depending on *d* and mode polarization, as summarized in Table 2.

**Figure 9.**
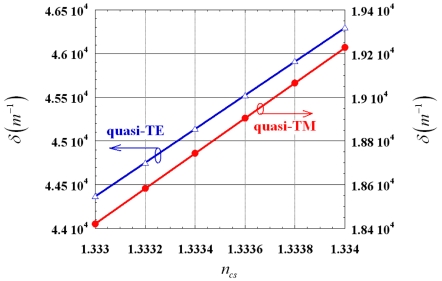
*δ* as a function of *n_cs_* for quasi-TE and quasi-TM modes.

Thus, it is clear that *δ* does not depend on *d* (being the second additive term in Eq. (10) negligible under previously discussed conditions), but it is only a function of slot waveguide geometrical parameters and *n_cs_*. For quasi-TE and quasi-TM modes, *δ* dependence on *n_cs_* is given in Figure 9.

For both polarizations, *δ* increases with increasing *n_cs_*, but the phase mismatch between the modes propagating in the coupler waveguides is significantly larger for quasi-TE than for quasi-TM polarization. Because of the linearity of *δ* dependence on *n_cs_*, we can write:

(15) $$\delta =p_{0}+p_{1}n_{cs}$$

where *p_0_* and *p_1_* are fitting parameters. It results *p_0_* = -2.53 μm^-1^ and *p_1_* = 1.932 μm^-1^ for quasi-TE and *p_0_* = -1.056 μm^-1^ and *p_1_* = 0.806 μm^-1^ for quasi-TM mode, respectively.

Combining Eq. (6) with Eq. (14) and (15), a new expression of normalized power coming out *W1* can be obtained:

(16) $$P_{1}\left(L,n_{cs}\right)=\Psi^{2}\frac{{\left(p_{0}+p_{1}n_{cs}\right)}^{2}+\left[{\left(q_{0}+q_{1}n_{cs}\right)}^{2}-{\left(p_{0}+p_{1}n_{cs}\right)}^{2}\right]cos^{2}\left[\left(q_{0}+q_{1}n_{cs}\right)L\right]}{{\left(q_{0}+q_{1}n_{cs}\right)}^{2}}$$

**Figure 10.**
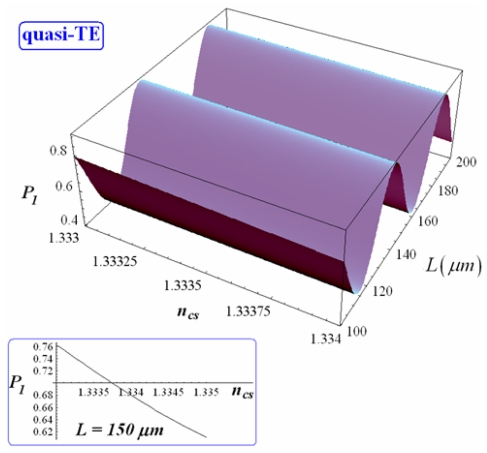
Normalized optical power exiting from *W1* and its dependence on *L* and *n_cs_* (quasi-TE, *d* = 1 μm). In the inset the dependence of *P_1_* on *n_cs_* is shown assuming *L* = 150 μm.

**Figure 11.**
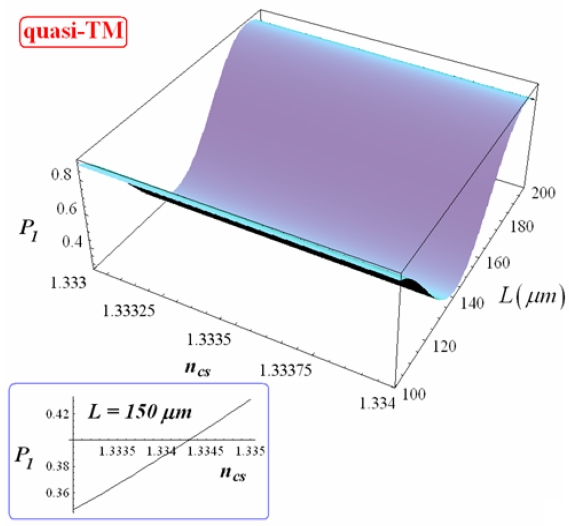
Normalized optical power exiting from *W1* and its dependence on *L* and *n_cs_* (quasi-TM, *d* = 1 μm). In the inset the dependence of *P_1_* on *n_cs_* is shown, with *L* = 150 μm.

Dependence of *P_1_* on *L* and *n_cs_* is shown in Fig. 10 and 11 for quasi-TE and quasi-TM polarization, respectively (with *d* = 1 μm and *Ψ*^2^ = 0.9).

As previously mentioned, *P_1_* oscillates as *L* varies. Oscillation rate is larger for quasi-TE mode than for quasi-TM one. As derived by Eq. (16), since it results (*q*_0_ + *q*_1_*n_cs_*) > (*p*_0_ + *p*_1_*n_cs_*) for each value of *d* and both polarizations (see Figures 8 and 9), the maximization of *P_1_* requires:

(17) $$cos^{2}\left[\left(q_{0}+q_{1}n_{cs}\right)L\right]=1\Leftrightarrow \left(q_{0}+q_{1}n_{cs}\right)L=m\pi$$

where *m* is an integer number.

**Figure 12.**
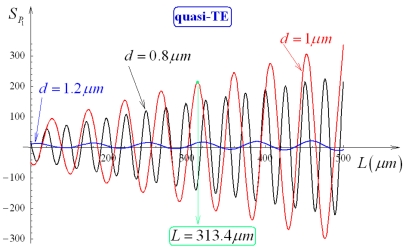
*S_P_*_1_ dependence on *L*, for *d* equal to 0.8 μm, 1 μm, 1.2 μm (quasi-TE, *Ψ*^2^ = 0.9).

**Figure 13.**
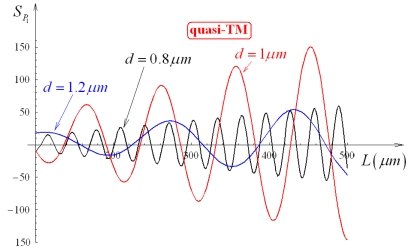
*S_P_*_1_ dependence on *L*, for *d* equal to 0.8 μm, 1 μm, 1.2 μm (quasi-TM, *Ψ*^2^ = 0.9).

Normalized power coming out *W1* has a linear dependence on *n_cs_* for each coupler length and both polarizations (see insets in Figures 10 and 11). The sensitivity of normalized power coming out of *W1* to *n_cs_* change can be defined as:

(18) $$S_{P_{1}}={\frac{\partial P_{1}}{\partial n_{cs}}|}_{n_{cs}=n_{cs}^{*}}$$

where $n_{cs}^{*}$ is the *n_cs_* reference value in which the derivative is calculated (we have assumed $n_{cs}^{*}=1.333$). This sensitivity is shown in Figures 12 and 13 as a function of *L*, for *d* = 0.8 μm, 1 μm, 1.2 μm and quasi-TE and quasi-TM polarizations, respectively. By comparing Figure 12 with 13, we can observe *S_P_*_1_ as significantly larger for quasi-TE than for quasi-TM mode and oscillating as *L* increases (oscillations are slower for quasi-TM than for quasi-TE). Values of relative maxima of *S_P_*_1_ progressively increase by increasing *L*, thus an arbitrary *S_P_*_1_ value could be obtained by simply increasing *L*. However, an appropriate trade-off between coupler sensitivity and length has to be usually achieved.

**Figure 14.**
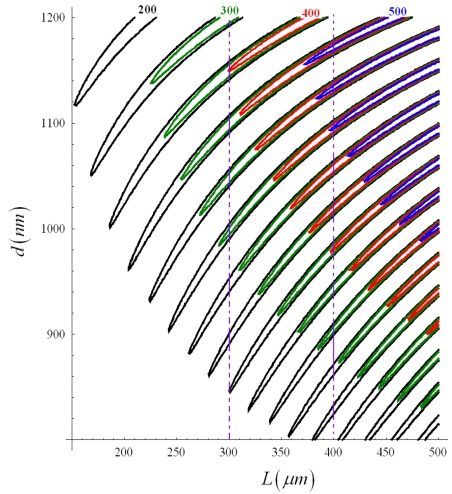
Level curves of *S_P_*_1_ as a function of *L* and *d* (quasi-TE mode, *Ψ^2^* = 0.9).

For *d* = 1 μm, larger sensitivity values can be obtained for either quasi-TE or quasi-TM modes. For example, assuming *d* = 1 μm and *L* = 313.4 μm, a sensitivity *S_P_*_1_ = 215.29 is obtained for quasi-TE mode. *S_P_*_1_ dependence on *d* and *L* has been investigated for quasi-TE mode by calculating *S_P_*_1_ level curves, as sketched in Figure 14.

**Figure 15.**
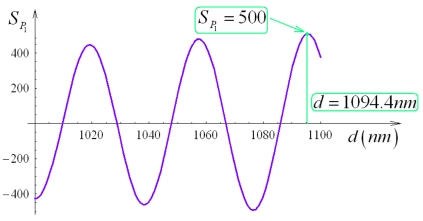
*S_P_*_1_ as a function of *d* for *L* = 400 μm (quasi-TE mode, *Ψ^2^* = 0.9).

Increasing both geometrical parameters (i.e. sensor total area), an improvement of sensitivity can be observed. Assuming for *S_P_*_1_ a target value of 500, we observe that this sensitivity value cannot be obtained for *L* = 300 μm, while becoming achievable for *L* = 400 μm. *S_P_*_1_ dependence on *d* has been investigated for *L* = 400 μm and quasi-TE polarization (see Figure 15), observing again an oscillatory behaviour. Minimum *d* value to achieve *S_P_*_1_ = 500 is equal to 1,094.4 nm. Geometrical parameters of the optimized sensor are summarized in Table 3.

In a directional coupler, the sum of normalized optical powers coming out from *W1* and *W2* is a constant value, *Ψ*^2^. Thus, the difference between *P_1_* and *P_2_* can be written as:

(19) $$P_{1}-P_{2}=P_{1}-\left(\Psi^{2}-P_{1}\right)=2P_{1}-\Psi^{2}$$

Therefore, the power difference sensitivity to the sensing waveguide cover index change is given by:

(20) $$S_{\left(P_{1}-P_{2}\right)}={\frac{\partial \left(P_{1}-P_{2}\right)}{\partial n_{cs}}|}_{n_{cs}=n_{cs}^{*}}=2S_{P_{1}}$$

Using the coupler sizes in Table 3, a value of *S*_(_*_P_*_1–_*_P_*_2)_ = 1,000 can be obtained.

This means that, if a (*P_1_-P_2_*) shift equal to 1% is detectable by the photo-detection apparatus, a change of *n_cs_* value of the order of 10^-5^ could be detected by a device having a length of 400 μm and a total area of 1,200 μm^2^. This value of minimum detectable refractive index is well five times lower than that reported for an integrated optical chemical sensor based on a conventional 10 mm long directional coupler [2], and about two times lower than that for a ring resonator-based optical biosensor, having a total area of 11,300 μm^2^ [9]. The integrated optical chemical sensor reported in [3] is very sensible, having a minimum detectable change of mode effective index as low as 10^-8^. However, this result is obtained by integrating a phase modulator within the sensor, i.e. at the expenses of device complexity and very large sensor dimensions (device area = 8×10^7^ μm^2^).

If the sensor designed in this paper is used to sense glucose concentration, its sensitivity should be around 0.1 g/L. Moreover, the theoretical minimum detectable refractive index in our architecture is more than one order of magnitude lower than that experimentally obtained value by the sensor reported in [51], having an area of 19,600 μm^2^ and adopting a Si_3_N_4_/SiO_2_ slot waveguide.

## 5. Conclusions

In this paper, we have proposed, modelled and optimized a directional coupler-based integrated optical chemical sensor. The coupler is formed by two nanometer-scale SOI slot waveguides. Monitoring the difference between optical power coming out from the two waveguides, it is possible to estimate with high accuracy the concentration of an analyte in the aqueous solution used as cover medium of one of the two waveguides forming the coupler. Sensor modelling is carried out by using the coupled mode theory formulation, while coupling coefficients between propagating modes and their phase mismatch are estimated by 2D full-vectorial finite element method. This modelling technique has been validated by comparing its results with those provided by the highly accurate 3D EME method.

An effective optimization procedure has been developed to obtain a very good trade-off between sensor dimensions and sensitivity. A minimum detectable refractive index change of the order of 10^-5^ can be obtained by a device having a very reduced occupation area, equal to 1,200 μm^2^. Because the photonic sensor is based on the use of an asymmetric directional coupler, read-out apparatus is expected to be very simple, i.e. to measure an analyte concentration shift it should be necessary to sense only an optical power change. If the sensor is used to estimate the glucose concentration in an aqueous solution, a resolution around 0.1 g/L can be theoretically predicted.

## Figures and Tables

**Table 1.** Parameters of coupled slot waveguides (effective indices, confinement factors and sensitivities calculated by assuming an aqueous solution as cover medium).

***Parameter***	***Value***
Si-wire height (*h*)	324 nm
Si-wire width (*w*)	180 nm
Slot gap region width (*g*)	100 nm
Effective index (quasi-TE)	1.578638
Effective index (quasi-TM)	1.999899
Cover confinement factor (quasi-TE)	0.7644
Cover confinement factor (quasi-TM)	0.4117
Waveguide sensitivity (quasi-TE)	1.0076
Waveguide sensitivity (quasi-TM)	0.4040

**Table 2.** Fitting parameters *q_0_* and *q_1_* for *d* = 0.8 μm, 1 μm, 1.2 μm.

	***d* = 0.8 μm**		***d* = 1 μm**		***d* = 1.2 μm**	

	**quasi-TE**	**quasi-TM**	**quasi-TE**	**quasi-TM**	**quasi-TE**	**quasi-TM**
***q****_0_***(μm^-1^)**	-0.5894	-0.0625	-1.5080	-0.5688	-2.1670	-0.9662
***q****_1_***(μm^-1^)**	0.5349	0.1228	1.1820	0.4514	1.6601	0.7398

**Table 3.** Geometrical and performance parameters of the optimized directional coupler for optical chemical sensing (slot waveguides parameters are in Table 1).

***Parameter***	***Value***
Coupler length (L)	400 μm
Distance between slot waveguides (d)	1,094.4 nm
Device area	1,200 μm^2^
*S_P_*_1_ (quasi-TE mode)	500
*S_(P_*_1–_*_P_*_2_*_)_* (quasi-TE mode)	1,000
Minimum detectable refractive index change	10^-5^
Minimum detectable glucose concentration	0.1 g/L
